# Retrograde Fixation of the Ulna in Pediatric Forearm Fractures Treated With Elastic Stable Intramedullary Nailing

**DOI:** 10.7759/cureus.8182

**Published:** 2020-05-18

**Authors:** Yvonne-Mary Papamerkouriou, Michail Christodoulou, Panayotis Krallis, Rohan Rajan, John Anastasopoulos

**Affiliations:** 1 Orthopaedics, Panagiotis & Aglaia Kyriakou Children's Hospital, Athens, GRC; 2 Orthopaedics, Attikon University Hospital, Athens, GRC; 3 Orthopaedics, Agia Sofia Children's Hospital, Athens, GRC; 4 Orthopaedics, Royal Derby Hospital, Derby, GBR

**Keywords:** retrograde, forearm fracture, paediatric, ulna, esin

## Abstract

Introduction

This study analyzes the outcomes of retrograde fixation of the ulna in pediatric forearm fractures treated with elastic stable intramedullary nailing (ESIN).

Materials and Methods

A retrospective analysis was conducted by reviewing patient records of forearm fractures treated with ESIN by retrograde fixation. The study included 30 children (26 boys and 4 girls). The mean age at the time of injury was 11.7 years (range: 6.6 to 14.3 years). The technique is described. All patients were followed up until hardware removal.

Results

The mean time for fracture healing was 5.3 weeks (range: 4 to 8.8 weeks). The mean time for nail removal was 6.6 months (range: 5 to 10 months). There were five cases with rotation deficits, one of which was a re-fracture.

Conclusions

When antegrade nailing is performed, the ulna is sometimes complicated by non-union as well as entry point irritation. We did not encounter such complications. Retrograde fixation of the ulna in pediatric forearm fractures treated with ESIN is a safe and effective alternative to common fixation (antegrade ulnar fixation) and offers technical advantages.

## Introduction

Forearm fractures constitute about 40% of pediatric fractures. The standard fixation for pediatric forearm fractures are closed reduction and casting [1]. The child’s physeal growth and remodelling potential allow the use of varying degrees of angulation, taking into account the child’s age. Surgical management is required only when conservative treatment has failed. Surgical treatment options include both plate fixation and elastic stable intramedullary nailing (ESIN) [2]. ESIN has been shown to have excellent clinical results and, in contrast to plate fixation, is considered a minimally invasive procedure [3,4]. Fracture fixation with flexible nails results in decreased surgical dissection and retention of biological factors at the fracture site [5,6].

The standard operative technique as described by Lascombes et al. includes retrograde nailing of the radius coupled with antegrade fixation of the ulna through the posterolateral cortex of the olecranon [7,8]. A problem related to antegrade fixation of the ulna is skin irritation over prominent ulnar hardware, sometimes requiring early removal of the ulnar pin [9]. In addition, delayed union or non-union of the ulna has been reported as a complication in a series of antegrade nailing. Ogonda et al. proposed a theory explaining the relation of delayed and non-union of the ulna to antegrade fixation [10].

We describe the operative technique of retrograde fixation of the ulna in forearm fractures treated with ESIN. We also report treatment outcomes and complications.

## Materials and methods

A retrospective analysis was performed of children with forearm fractures treated at our institution with ESIN by retrograde fixation of both bones during a period of six years. These fractures occurred in otherwise healthy children. Pathological fractures or forearm fractures in children with underlying neuromuscular disorders or metabolic disorders, as well as Monteggia or Galeazzi fractures were excluded. The study was approved by the Ethics Committee of our hospital, and all parents gave informed consent.

There were 30 patients (26 boys and 4 girls), with a mean age of 11.7 years (range: 6.6 to 14.3 years). The mean age of male patients was 11.9 years and that of females was 9.8 years. Left forearm fractures were more frequent than right ones, with a ratio of 1 right to 1.7 left (11 right:19 left). One patient had an associated injury of a concomitant palmar deep laceration that was explored and sutured in the operating room. Three patients presented with open fractures: two were Gustilo I and one was a Gustilo II open fracture. The most common mechanism of injury was a fall onto an outstretched hand from the patient’s own height. One patient was injured during a motor vehicle accident, and another fell off a tree. Two patients were injured in a playground environment, more specifically one fell off a monkey bar and another was injured falling off a trampoline. Four injuries were sports-related.

Indications for the surgical procedure were unacceptable closed reduction or secondary loss of reduction seen at follow-up. Five certified orthopedic surgeons were responsible for these procedures, and, in almost half of the cases, the surgery was performed by orthopedic trainees under their supervision. A fracture table was used, and fluoroscopic control was available for each case. A tourniquet was applied in every procedure. Titanium elastic nails of either 2 or 2.5 mm diameter were used depending on the medullary isthmus so that the nail’s diameter was at least two-thirds the width of the narrowest part of the medullary canal. Both bones were fixed in a retrograde fashion. Radial nailing was performed through a skin incision of no more than 2 cm on the lateral edge of the radial distal metaphysis, and the entry point was approximately 2 cm proximal to the distal physis. Likewise, ulnar nailing was performed through a skin incision of a maximum of 2 cm on the lateral edge of the ulnar distal metaphysis, starting at 3 cm from the palpable ulnar styloid, and the entry point was at approximately 2 cm proximal to the distal physis. In most cases, the radius was nailed first. The cut nail tips were bent at 90° or less and in few cases not at all. After surgery, the forearm was immobilized in an above-elbow full synthetic cast, and plain radiographs were obtained.

**Figure 1:**
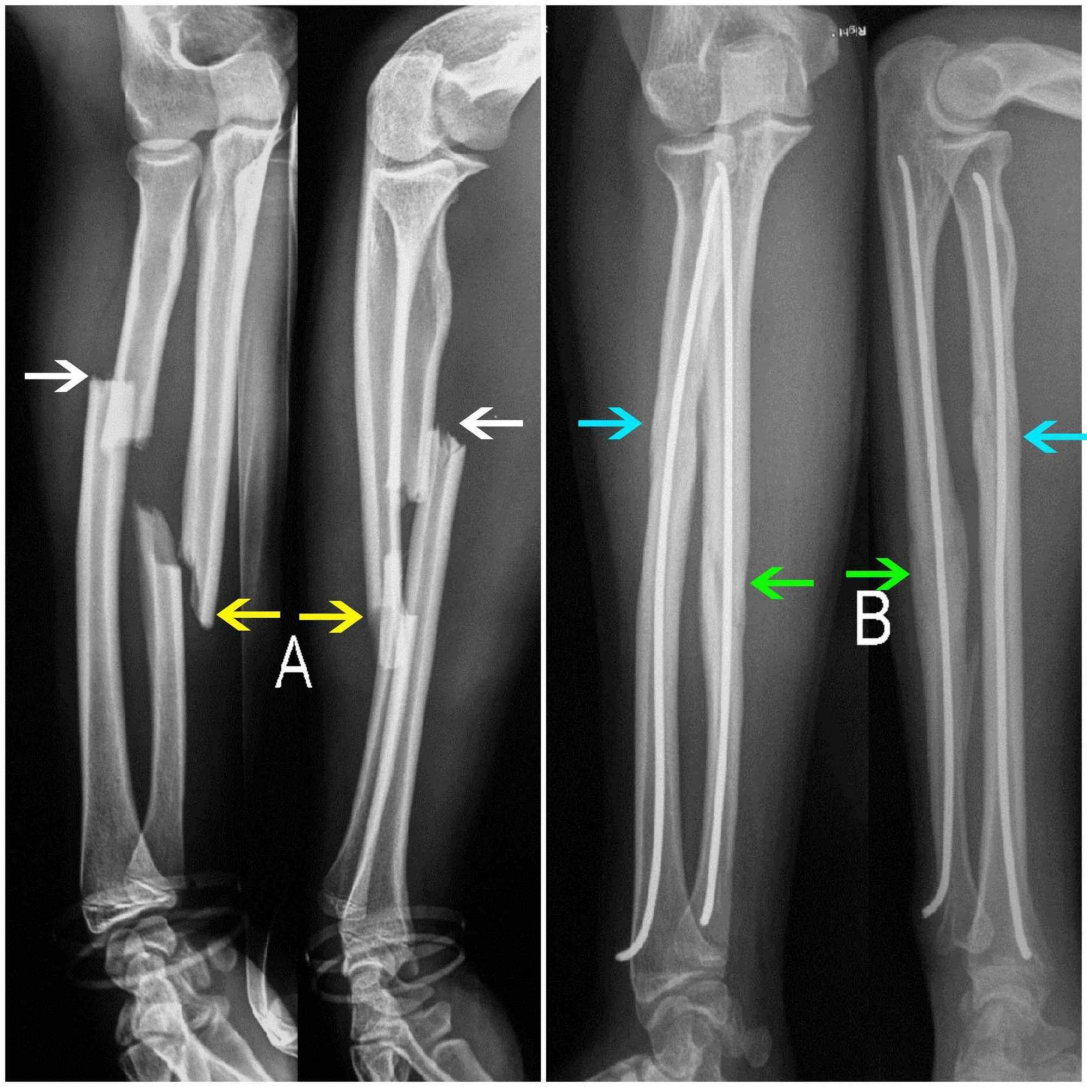
Initial fracture and four-month follow-up (A) Initial X-ray showing fracture of both the radius (white arrows) and the ulna (yellow arrows) in the anteroposterior (AP) and the lateral (L) view. (B) Four-month follow-up X-ray showing healing of both the radius (blue arrows) as well as the ulna (green arrows) in the AP and the L view.

On the first follow-up at one-month postoperative, the cast was removed and a new radiograph evaluation was performed. At that time, the patients were encouraged to start active mobilization of both the elbow and wrist joints. For those patients whose X-rays did not reveal complete healing at the one-month follow-up, a new review was arranged two months postoperatively. The healing process was determined on the basis of the clinical examination and two projection radiographs. Healing was considered sound when a bony bridge of at least three cortices was seen clearly on a plain radiograph in two projections. All patients were then re-assessed four months after surgical treatment, both clinically and by plain radiographs, in order for hardware removal to be scheduled (Figure 1). Hardware removal was not planned earlier than five months postoperatively. All patients were followed up at least until hardware removal.

Medical records were reviewed to assess the individual demographics and fracture characteristics. We recorded the mechanism of injury, any associated injuries, time to surgical procedure, the size of the nails used, postoperative hospital stay, and pre-existing as well as postoperative complications. Postoperative angulation at the fracture site was recorded in anteroposterior (AP) and lateral X-ray views. Nail tip prominence was measured as the maximum transverse distance from the side of each bone to the distal tip of the nail on an AP view, and the degrees of bending, if any, of the prominent nail tips were noted.

At the time of final follow-up, a careful clinical assessment was conducted recording remaining deficits in range of motion of the wrist (flexion and extension), forearm (pronation and supination), and elbow (flexion and extension) in comparison with the un-fractured forearm. Any residual angulation was calculated, as well as in patients with remaining rotation disturbances.

Maximum radial bowing was calculated on an AP radiograph of the forearm. The length of the radius (y) is measured from the bicipital tuberosity to the distal radioulnar joint. At the point of the maximum radial bow, a perpendicular line (r) is drawn to (y) and the distance (x) to this line is measured. To determine the site of the maximum radial bow, the distance from the bicipital tuberosity to the point of the maximum bow (x) is divided by the length of the entire bow (y) and expressed as a percentage (x/y x 100). Firl and Wünsch proposed that the maximum bowing should be below 10% of the radial length (mean value: 7.21%) [11].

## Results

**Figure 2:**
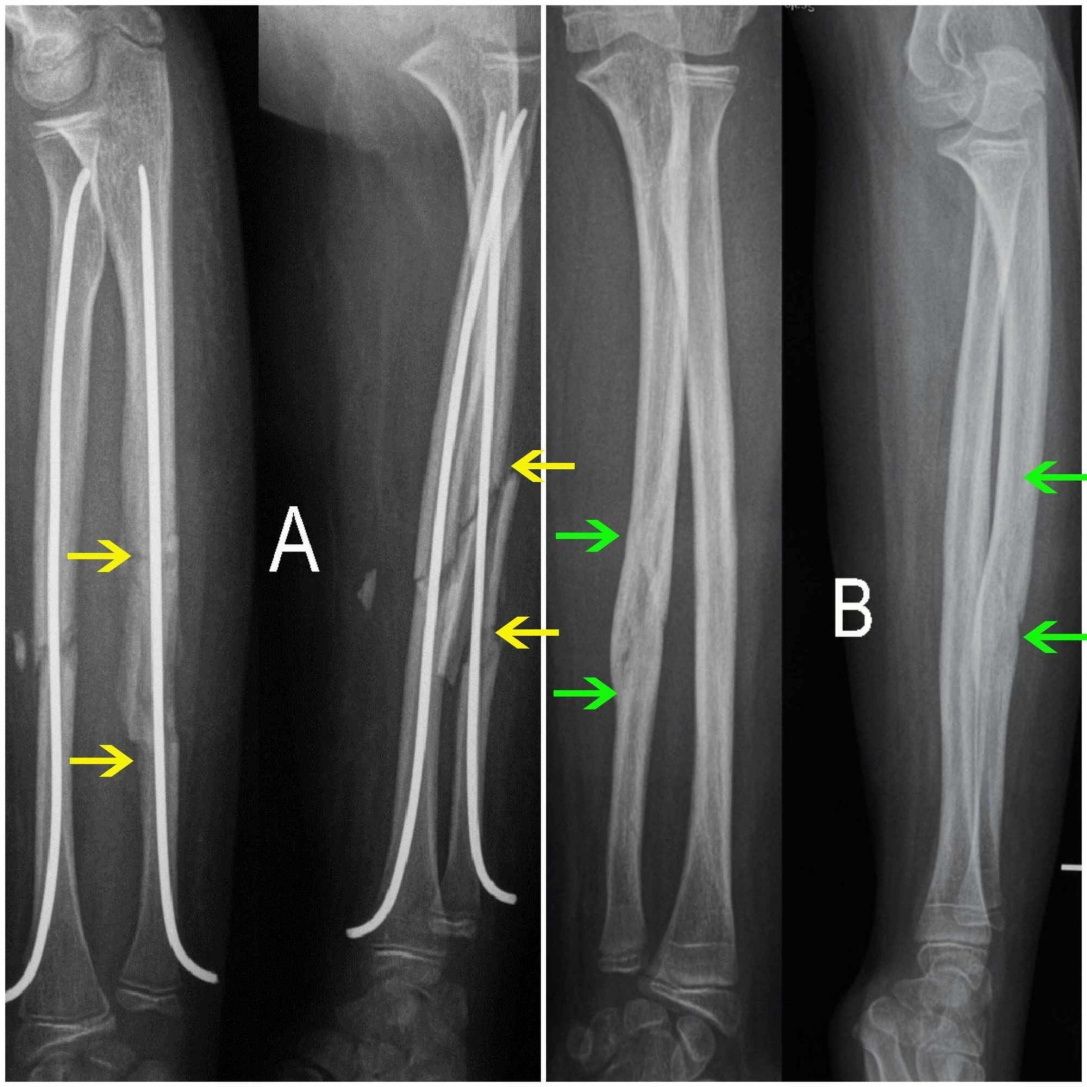
Ulnar segmental fracture Postoperative anteroposterior (AP) and lateral (L) X-ray of ulnar segmental fracture (yellow arrows) in both bone forearm fracture treated by ESIN. (B) Final follow-up AP and L X-ray after hardware removal showing complete healing of the ulnar fracture (green arrows), which is hardly evident anymore.

The most common fracture pattern was a transverse radius and ulna fracture. There was a single case of an oblique fracture pattern of the radius and ulna and another of an ulnar segmental mid-diaphyseal fracture in combination with a transverse radial mid diaphyseal fracture (Figure 2). Predominant location of fracture was that of the mid-diaphysis, with six cases having a combination of proximal radial with a mid-diaphyseal ulnar fracture.

Twenty-two patients were operated either on admission day, which coincided with the day of injury or on the next day. Eight patients were operated on between 7 and 16 days post-initial injury, after the loss of reduction following an initial attempt at conservative treatment. Out of a total of 60 fractures, open reduction was performed in 32. Mean postoperative hospitalization was 2.7 days (range: 1 to 5 days).

The mean value of nail protrusion lengths was 0.64 cm for the radius and 0.61cm for the ulna. The majority of tip bending was between 0° and 90°, with most bent just below 90°. However, a few nails were not bent. The mean time of fracture healing was 5.3 weeks (range: 4 to 8.8 weeks). Nail removal time was at a mean 6.6 months (range: 5 to 10 months), while in one case, removing an ulnar nail was impossible as it was cut too short to be removed. None of the nails was removed earlier than five months after the initial operation. Patient demographics and results are summarized in Table 1.

**Table 1:** Patient demographics and results M, male; F, female; R, right; L, left

Results	
Age at fracture	11.7 years (range: 6.6-14.3 years)
Gender, M:F	26:4
Side of fracture, R:L	11:19
Associated injuries	Left palmar laceration(1), Gustilo I (2), Gustilo II (1)
Fracture healing time	5.3 weeks (range: 4-8.8 weeks)
Nail removal time	6.6 months (range: 5-10 months)

At the final follow-up, the majority of patients had a full 90/90 (pronation-supination) range of motion. In both bone fractures of the forearm, residual angulation can lead to disruption of joint movement [12]. In five of the patients, pronation and/or supination deficits were recorded at the final follow-up.

**Figure 3:**
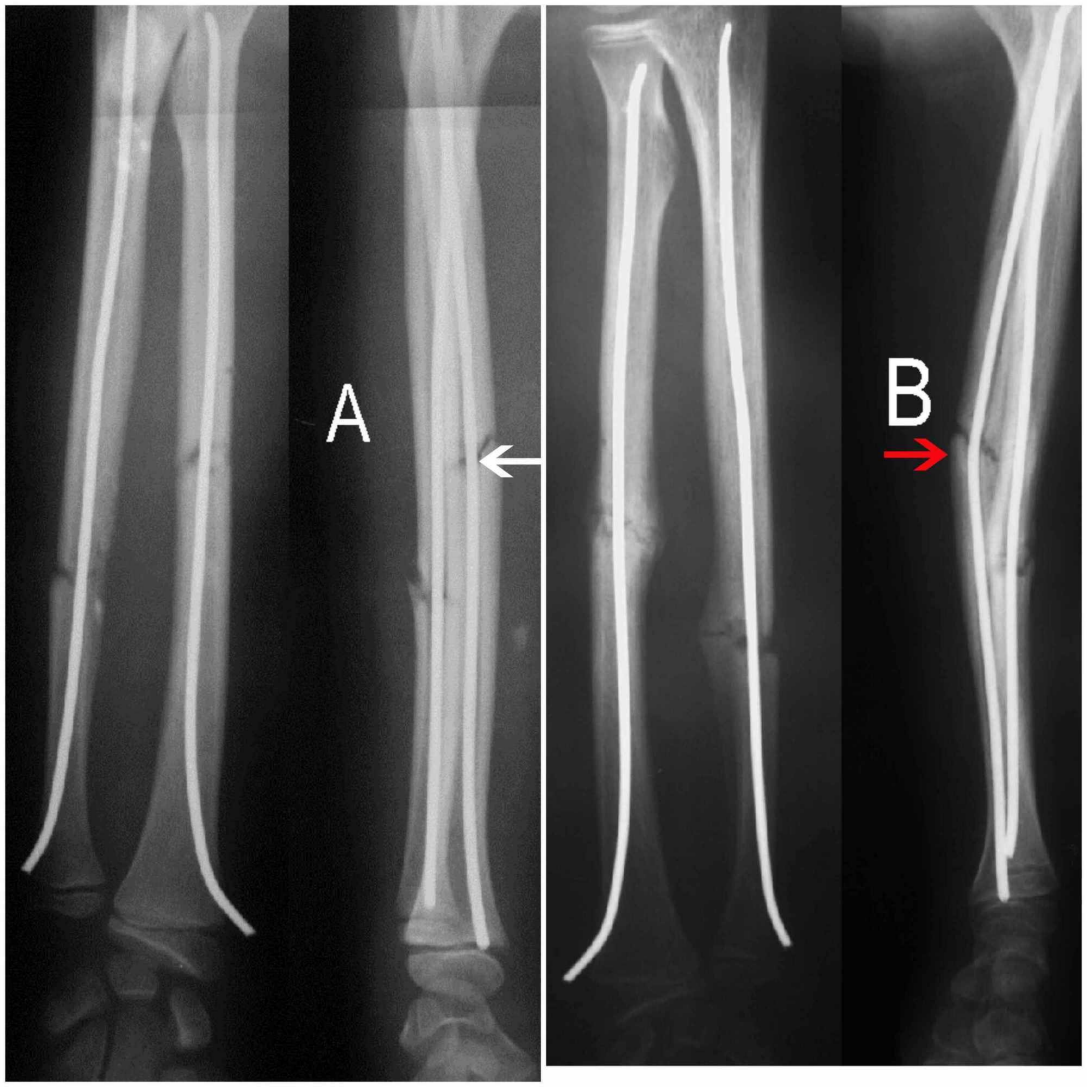
Angulation due to bending of the nail (A) Immediate postoperative X-ray showing acceptable reduction of the radial fracture and no angulation (white arrow) evident in the lateral (L) view. (B) One-month postoperative X-ray showing angulation of the radial fracture due to bending of the nail (red arrow) evident in the L view, in a case where no cast was used, and the patient suffered a new injury.

The first patient had no angulation postoperatively; however, angulation was noted on final follow-up X-rays, which could have been prevented. In this patient, no cast or splint was placed postoperatively (Figure 3). Unfortunately, the patient suffered a new injury during the early stages of healing, leading to bending of the radial nail and angulation of the fracture.

The second patient had nails that were too thin, leading to the production of immediate postoperative angulation.

In the third patient, there was no angulation at the final follow-up, and function was normal; however, the patient suffered a re-fracture six months after hardware removal. The re-fracture was treated conservatively by casting. This patient had the worst pronation deficit of 45° at follow-up, even though there was only slight angulation, in particular, less than 10° posterior angulation of the ulna and 10° ulnar angulation of the radius. The maximum radial bowing was 4% of the total radial length, well below the mean value of 7.21% as stated by Firl and Wünsch [11].

In the fourth patient, there was no evident angulation postoperatively nor at the final follow-up; however, the patient had a pronation deficit of 30°. This patient had a maximum radial bowing of 7.4% of total radial length, a little higher than the mean value according to Firl and Wünsch [11].

Similarly, in the fifth patient, there was no evident angulation either postoperatively or at the final follow-up; however, a pronation deficit of 10° was noted, and a high maximum radial bowing of 8% of the total radial length was measured, as summarized in Table 2.

**Table 2:** Rotation deficits

Pronation/supination deficit	Angulation postoperatively	Angulation at the final follow-up	Maximum radial bowing	Comments
20°/–	None	12° anterior radius, 10° anterior ulna	6%	No cast used postoperatively, new injury
10°/–	10° anterior radius	10° anterior radius	6.3%	Too thin nails used
45°/–	None	10° posterior ulna, 10° ulnar of the radius in healing after re-fracture	4%	Angulation present after re-fracture treated conservatively
30°/10°	None	None	7.4%	
10°/–	None	None	8%	

Stiffness can occur even with normal radiographs, and this could be indicative of fibrosis of the interosseous membrane and/or contracture of the interosseous ligament [13,14].

## Discussion

The ultimate goal when treating forearm fractures should be a good functional result whether treated surgically or conservatively. It has been demonstrated that 15° to 20° of angulation in the middle third forearm can lead to a major loss of forearm rotation [3]. This, however, does not necessarily result in a bad functional outcome. The exact amount of acceptable angulation, rotation, and displacement are controversial in the existing literature. Price recommends an acceptable angulation of under 15º in mid-shaft and distal shaft fractures, under 10º in the proximal shaft, under 30º of malrotation, and up to 100º displacement in children under eight years old [15].

Our cases consisted of unacceptable closed reduction or secondary loss of reduction. Surgical treatment was never chosen based solely on potential instability or the patient’s age. 

Retrograde nailing of the ulna was performed, which provides technical advantages in comparison to antegrade. The surgeon is not required to change his position with respect to the fracture table as retrograde fixation is performed for both bones. Furthermore, there is always good visualization of both nails and their progression on the image intensifier without moving the forearm.

Entry point complications

Complications related to the entry point in cases where antegrade nailing of the ulna is performed are perhaps the most common. In a large series of 553 children, in 5 cases, the hardware had to be removed prematurely because the skin was perforated by the osteosynthesis material [16]. In addition, bursitis developed in 14 children at the nail ends. In a retrospective study comparing plating with ESIN, complications included four cases of symptomatic hardware and one case of ulnar bursitis [17]. A recently published series of antegrade nailing of the ulna in forearm fractures reports skin irritation over prominent ulnar hardware in 9 children out of 88, leading to the early removal of the ulnar pin in one case [9]. In our series, there were no complications connected to the entry point of the nails.

Non-union of the ulna

A more significant complication of antegrade ulnar nailing is delayed union or non-union. Ogonda et al. describe three cases of closed fractures of both forearm bones in children treated by intramedullary nailing that were complicated by delayed union of the ulna in two patients and ulnar non-union in one patient [10]. The authors performed a limited review of the literature, recording cases of delayed union [18-20]. In a retrospective study of 25 patients, a delayed union of an open grade 1 fracture of the ulna is described [21].

In an attempt to explain why, in their experience, the ulna is the bone complicated by non-union, Ogonda et al. argue that the antegrade surgical technique utilized for insertion of the ulnar nail could be responsible. The medullary canal of the distal ulna narrows considerably compared with that of the proximal radius. Hence, the curved tip of the nail, which is relatively rigid in comparison with the body of the nail, encounters more resistance against the walls of the narrow medullary canal of the distal ulna as it is advanced distally. This causes distraction at the ulnar fracture site. Ogonda et al.’s hypothesis was dismissed by Schmittenbecher et al. because of no evident distraction seen on X-rays. They, nevertheless, also reported the ulna to be mostly affected by delayed union as, in a multicenter study of 532 patients, seven cases of delayed union involved the ulna, whereas only three involved the radius [22]. In a retrospective analysis of 553 children with forearm shaft fractures treated with ESIN, 7 cases of ulnar non-union were recorded and 14 patients had delayed union, out of which 13 suffered from delayed union of the ulna and only two of the radius [16]. Smith et al., comparing operative techniques in 21 children treated by ESIN, found two delayed unions and one non-union of the ulna [17]. In our series, there were no delayed unions or non-unions.

Superficial radial nerve complications

Lesions of the superficial radial nerve are a common complication in forearm shaft fractures treated with ESIN [7,23,24]. In a multicenter study of 553 children with forearm fractures, there were 13 children with hypoesthesia [16]. In a smaller series of 45 children, there were 3 children with hypoesthesia [25]. Due to the problems with the superficial radial nerve, the dorsal approach of Lister’s tubercle had been suggested [26]. In our series, this complication did not occur. We propose that a lateral approach accompanied by a blunt preparation of the entry point suffices in order to avoid this complication.

Dorsal sensory branch of ulnar nerve complications

In a recent paper with tips on avoiding complications after intramedullary nailing of forearm fractures, it is recommended that when performing retrograde fixation of the ulna, care should be taken to avoid the dorsal sensory branch of the ulnar nerve [27]. This is possible when taking into account the anatomical features of the nerve. The dorsal cutaneous branch of the ulnar nerve arises from the main ulnar nerve an average of 5.5 cm proximal to the head of the ulna [28]. In our series, there were no ulnar nerve complications.

## Conclusions

Retrograde fixation of the ulna in pediatric forearm fractures treated with ESIN is a safe and effective method of treatment. In addition, it provides technical advantages such as the same position of surgeon throughout the procedure and good visualization of both nails on the image intensifier. Delayed union or non-union of the ulna, as well as entry point complications, did not occur in our series. This method is an adequate alternative to antegrade ulnar fixation and offers practical assets.
